# Using Iterative RE-AIM to enhance hospitalist adoption of lung ultrasound in the management of patients with COVID-19: an implementation pilot study

**DOI:** 10.1186/s43058-022-00334-x

**Published:** 2022-08-12

**Authors:** Anna M. Maw, Megan A. Morris, Russell E. Glasgow, Juliana Barnard, P. Michael Ho, Carolina Ortiz-Lopez, Michelle Fleshner, Henry R. Kramer, Eric Grimm, Kate Ytell, Tiffany Gardner, Amy G. Huebschmann

**Affiliations:** 1 Division of Hospital Medicine, University of Colorado School of Medicine, Aurora, USA; 2 Adult and Child Consortium for Health Outcomes Research and Delivery Science (ACCORDS), University of Colorado School of Medicine, Aurora, CO 80045 USA; 3 Division of Cardiology, University of Colorado School of Medicine, Aurora, CO 80045 USA; 4 Internal Medicine Residency Program, University of Colorado School of Medicine, Aurora, CO 80045 USA; 5 Division of General Internal Medicine, University of Colorado School of Medicine, Aurora, CO 80045 USA

**Keywords:** Implementation science, COVID-19, Lung ultrasound, RE-AIM

## Abstract

**Background:**

Lung ultrasound (LUS) is a clinician-performed evidence-based imaging modality that has multiple advantages in the evaluation of dyspnea caused by multiple disease processes, including COVID-19. Despite these advantages, few hospitalists have been trained to perform LUS. The aim of this study was to increase adoption and implementation of LUS during the 2020 COVID-19 pandemic by using recurrent assessments of RE-AIM outcomes to iteratively revise our implementation strategies.

**Methods:**

In an academic hospital, we implemented guidelines for the use of LUS in patients with COVID-19 in July 2020. Using a novel “RE-AIM dashboard,” we used an iterative process of evaluating the high-priority outcomes of Reach, Adoption, and Implementation at twice monthly intervals to inform revisions of our implementation strategies for LUS delivery (i.e., Iterative RE-AIM process). Using a convergent mixed methods design, we integrated quantitative RE-AIM outcomes with qualitative hospitalist interview data to understand the dynamic determinants of LUS Reach, Adoption, and Implementation.

**Results:**

Over the 1-year study period, 453 LUSs were performed in 298 of 12,567 eligible inpatients with COVID-19 (Reach = 2%). These 453 LUS were ordered by 43 out of 86 eligible hospitalists (LUS order adoption = 50%). However, the LUSs were performed/supervised by only 8 of these 86 hospitalists, 4 of whom were required to complete LUS credentialing as members of the hospitalist procedure service (proceduralist adoption 75% vs 1.2% non-procedural hospitalists adoption). Qualitative and quantitative data obtained to evaluate this Iterative RE-AIM process led to the deployment of six sequential implementation strategies and 3 key findings including (1) there were COVID-19-specific barriers to LUS adoption, (2) hospitalists were more willing to learn to make clinical decisions using LUS images than obtain the images themselves, and (3) mandating the credentialing of a strategically selected sub-group may be a successful strategy for improving Reach.

**Conclusions:**

Mandating use of a strategically selected subset of clinicians may be an effective strategy for improving Reach of LUS. Additionally, use of Iterative RE-AIM allowed for timely adjustments to implementation strategies, facilitating higher levels of LUS Adoption and Reach. Future studies should explore the replicability of these preliminary findings.

**Supplementary Information:**

The online version contains supplementary material available at 10.1186/s43058-022-00334-x.

**Contributions to the literature**

- While implementation science has shown considerable promise in enhancing the translation of research to practice, techniques to make this translation process more rapid are still needed
- In a multi-faceted implementation strategy termed “Iterative RE-AIM,” we periodically engaged hospitalists to identify emerging and persistent barriers to lung ultrasound implementation for the management of patients with COVID-19, thereby facilitating timely revisions to our other implementation strategies
- These findings add to the emerging literature on Iterative RE-AIM, suggesting it is a highly feasible and promising approach to accelerate the speed of implementation when delivered using tools such as the frequently updated RE-AIM dashboard.

## Introduction

Point of care lung ultrasound (LUS) is an ultrasound of the lung acquired and interpreted by a clinician at the bedside. Over the last two decades, there has been increasing interest in the integration of LUS into the assessment of dyspneic patients, as it has been shown to be more accurate than standard tests including physical exam maneuvers [1] and chest x-ray (CXR) for the many of the most common causes of dyspnea: pneumonia [2], pleural effusion [3], pulmonary edema [4], and pneumothorax [5]. Its use has also been shown to reduce urgent visits and hospitalization as well as improve quality of life in patients with heart failure [6–8]. LUS is now a recommended test by multiple professional societies guidelines [9–11]; however, it is still not widely used in clinical practice.

With the onset of the COVID-19 pandemic, LUS garnered additional interest from the clinical community due to its superior accuracy [8] and operational advantages over CXR, including conservation of personal protective equipment and reduced risk of nosocomial transmission. This is because, unlike CXR or computed tomography (CT), LUS is an evidence-based intervention that can be performed at the bedside by the treating clinician, obviating the need for transportation or involvement of a radiology technologist.

One of the barriers to realizing the advantages of LUS is that few hospitalists—the clinicians caring for a large proportion of patients with COVID-19 in the United States [12]—are trained in its use. The discipline of implementation science emerged out of a recognized need to accelerate the uptake of evidence-based interventions, like LUS, into clinical and public health practice [13, 14]. One of the challenges to achieving this goal is developing methods for acquiring timely and actionable data on the progress of implementation outcomes, so that other strategies can be employed if current approaches are not advancing intervention uptake [15, 16]. In order to address this research methods gap as well as speed the integration of LUS into practice, we used an approach titled Iterative RE-AIM [17]. Iterative RE-AIM is a relatively new application of the RE-AIM framework [17] which was initially developed to promote external validity and equity in research of health interventions by measuring both implementation *and* effectiveness outcomes [18]. By evaluating the progress of implementation at regular intervals, Iterative RE-AIM allows for data-driven mid-course adjustments in implementation strategies, with the goal of achieving improved implementation.

The aim of this study was to better understand the feasibility of employing the Iterative RE-AIM process to recurrently assess the implementation outcomes of Reach, Adoption, and Implementation to inform planned adaptations to implementation strategies during this pilot study focused on LUS implementation for the management of patients with COVID-19 during the 2020 COVID-19 pandemic.

## Methods

### Conceptual framework

As mentioned, Iterative RE-AIM is a relatively new application of the RE-AIM framework [17], which was initially developed to promote external validity and equity in research of health interventions by measuring both implementation *and* effectiveness outcomes [18]. The RE-AIM domains include Reach, Effectiveness, Adoption, Implementation, and Maintenance. This 1-year pilot study was designed to focus on the RE-AIM outcomes of Reach, Adoption, and Implementation that were of particular priority to our hospitalist partners. Following pragmatic use of RE-AIM [19] and given this was a short-term pilot study, Effectiveness and Maintenance were not evaluated. During the implementation phase, we engaged hospitalists to understand how and why the LUS intervention was or was not being used, thus helping to inform and identify implementation strategies to yield a better fit between intervention and setting, ultimately seeking to improve RE-AIM outcomes.

### Study setting and participants

From July 2020 to June 2021, we conducted a single arm implementation pilot study in a quaternary academic medical center in Aurora, CO. During the period of data collection, there were 15 hospitalist-run services caring for approximately 200 patients daily, including 2 dedicated to the care of patients with COVID-19, as well as a procedure service. The procedure service was a consultative service that performed bedside procedures and diagnostic point of care ultrasound (POCUS) studies, including LUS, at the request of other hospitalist services. The procedure service attendings consisted of a core faculty group of 10 hospitalists who were experts in performing bedside procedures such as paracentesis, thoracentesis, central venous access, and lumbar puncture.

During the one-year study period, two hospitalist faculty received salary support through NIH/NCATS Colorado CTSA project grant #UL1 TR002535 to train other hospitalists in LUS. All 90 clinicians within the Division of Hospital Medicine (DHM), both physicians and advanced practice providers (APP), contributed data to this study. At the start of the study, only 4 DMH clinicians were credentialed in LUS while 86 were eligible to undergo training, complete credentialing, and subsequently adopt the independent performance of LUS in their care of patients. Hospitalists were made aware of the study during Hospital Medicine Division meetings. Emails containing information about the study as well as a postcard consent were sent to all hospital medicine faculty. There were no commitments to training or ordering LUS contained within the consent.

At the time of data collection, there were no professional society guidelines regarding indications for LUS in patients with COVID-19. All patients hospitalized on the hospitalist services positive for COVID-19 were considered eligible for LUS as study investigators considered a baseline LUS exam a potentially diagnostically useful comparison study should the patient’s respiratory status worsen later in the hospitalization. Data collection occurred from July 2020 through June 2021. This study was approved by the University of Colorado Multiple Institutional Review Board (COMIRB) in May 2020.

### Intervention

We consider the evidence-based practice of LUS to be a complex intervention as it requires performance of several sequential steps or core components to impart patient benefit. Accordingly, per recent guidance offered by Perez Jolles et al., we define fidelity as the degree to which the core function is maintained while allowing for adaptations in form [20]. We consider the core functions of our intervention to include the (1) acquisition of LUS images that were adequate in quality based on professional society standards [21], (2) image interpretation according to expert recommendations [22], and (3) clinical decision-making by the patient’s clinical team, incorporating the LUS image interpretation and their clinical judgment. Fidelity was determined by assessment of these 3 core functions via image and chart review by LUS (expert) faculty as part of usual clinical operations to ensure the quality of LUS use.

### Initial implementation strategies

**Fig. 1:**
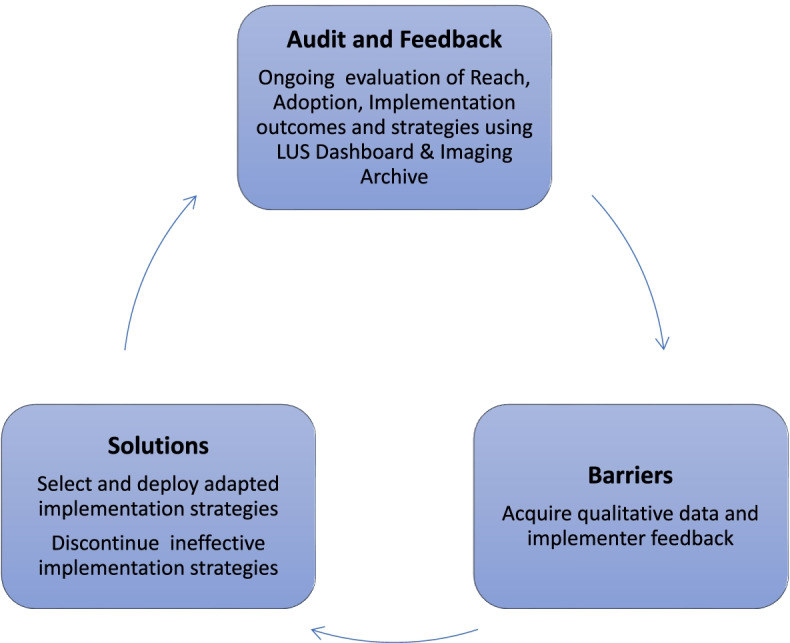
Iterative RE-AIM process to revise the LUS implementation strategies used

Implementation strategies are approaches used to facilitate uptake of an intervention, targeting known or anticipated barriers to implementation at multiple levels of the context in which implementation efforts are occurring [23]. The Expert Recommendations for Implementing Change (ERIC) has compiled 73 distinct implementation strategies [23]. We employed 3 initial strategies that we refer to according to their ERIC category—these included a LUS training program (ERIC strategy of conducting ongoing training), a RE-AIM dashboard (ERIC strategy of audit and feedback), and the Iterative RE-AIM process (see Fig. 1) of audit and feedback by reviewing the RE-AIM dashboard and revising implementation strategies (considered a package of ERIC strategies, including “organizing implementation team meetings” and “tailoring strategies”) based on qualitative feedback from hospitalists. Each of these implementation strategies is described further below.

#### LUS training

All DHM physicians and APPs were eligible for training in LUS. Training included a 1-h didactic, submission of a 25 LUS exam portfolio in which image acquisition, image interpretation, and clinical decision-making were assessed, and a proctored scanning session conducted by a faculty member with hospital privileges in LUS. Once credentialed, faculty were granted hospital privileges to perform LUS in patients independently and to bill for exams. All images used for patient care were uploaded to an archive and could be viewed by clinicians through a link in the EMR. Clinicians were required to document their LUS interpretation and clinical decision-making in the EMR.

#### Iterative RE-AIM process with the use of a RE-AIM dashboard innovation

The overarching implementation strategy used in this pilot study was an Iterative RE-AIM process using audit and feedback described as 3 steps (see Fig. 1): Step (1) RE-AIM outcomes were evaluated by the implementation team, consisting of 4 hospitalists, using the RE-AIM dashboard and POCUS imaging archive; Step (2) Barriers to progress were explored with hospitalist primary adoptors; and Step (3) Implementation strategies informed by participating staff perspectives were selected and deployed by the implementation team to address current barriers. Iterative RE-AIM has been described previously as a feasible strategy to promote planned adaptations at multiple timepoints *during* the implementation phase to enhance implementation in a context-sensitive and timely manner [17]. Implementation strategies were selected through discussion and consensus among members of the implementation team, with consideration of quantitative RE-AIM outcomes and discussions of preliminary themes emerging from the hospitalist interviews.

**Fig. 2:**
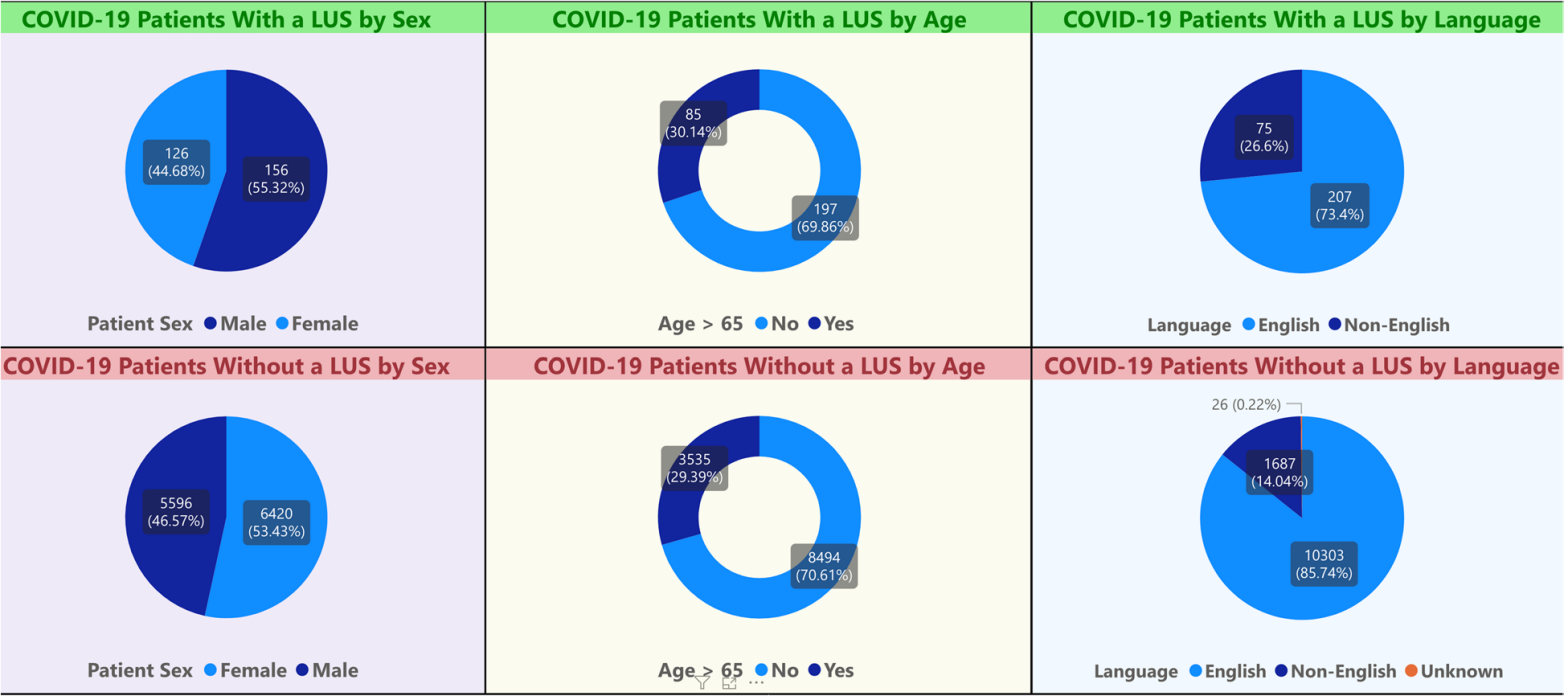
RE-AIM dashboard compares the representativeness of LUS Reach by sex, age, and language spoken

To obtain the quantitative data for the Iterative RE-AIM process, we worked with the DHM analytics team to create a virtual RE-AIM dashboard [24] (Fig. 2). Development of the dashboard required approximately 80 h of time for a master level health informaticist to build, but minimal time to maintain over the course of the study. The dashboard displayed RE-AIM outcomes by automatically extracting quantitative data from the electronic health record (EHR) every 48 h (Fig. 2). This provided nearly real-time audit and feedback [23]. As access to operational dashboards that extract EHR data are generally kept secure due to the presence of personalized clinician practice information, the PI accessed RE-AIM outcomes displayed on the dashboard on behalf of the implementation team, just prior to the twice monthly project meetings, as a means of monitoring implementation progress and screening for implementation barriers. The PI then reported the dashboard findings to the other members of the implementation team at project meetings. The twice monthly project meetings, during which members of the implementation team convened, were considered part of over-arching iterative RE-AIM strategy. The implementation team consisted of the PI, who is a member of the LUS hospitalist faculty, and 3 additional LUS hospitalist faculty. The team would then discuss the RE-AIM dashboard data as well as the contextual factors related to the less than optimal RE-AIM domain results. This information was then used to inform the selection of new implementation strategies and the de-implementation of strategies that were deemed ineffective. Concurrent qualitative data were collected, through interviews with DHM faculty, to understand facilitators and barriers to adoption. Methods used to acquire these qualitative data have been described previously [25].

### Measures and study procedures

As mentioned above, per our implementation partners’ recommendation, the RE-AIM domains measured as part of our Iterative RE-AIM process included Reach, Adoption, and Implementation. Reach was measured as the percentage of eligible patients hospitalized with a positive COVID-19 test result who received a LUS during the hospitalization. As recommended by Glasgow et al. [26], to assess the representativeness (equity) of Reach, we compared the demographics and characteristics of eligible patients who received a LUS to those who did not, in order to identify disparities in Reach that could be addressed by adapting our implementation strategies. Adoption was measured by calculating the percentage of clinicians who had completed the credentialing process, been granted hospital privileges to perform LUS, and performed at least one LUS exam as a credentialed clinician.

Implementation was assessed on an ongoing basis including measures of fidelity and adaptation. Fidelity was measured using data available through the POCUS imaging archive and EHR. LUS exams reviewed were gathered by random selection of at least 10% of LUS exams within the EHR. Determination of fidelity was measured using a standardized process by which image quality, image interpretation, and clinical decision-making were assessed by LUS faculty via review of the POCUS imaging archive and EHR documentation. Adaptations were identified and discussed at the twice monthly implementation meetings as part of the Iterative RE-AIM process and were categorized in the following matter: spontaneous (i.e., change in area of lung imaged) versus planned (i.e. deployment of implementation strategy); fidelity consistent (i.e., change in ultrasound video clip length from 6 secs to 8 secs) versus inconsistent (i.e., change in the number of areas of lung imaged from 6 areas to 1 area) [27]; and level of adaptation: intervention (i.e., decreasing the number of images acquired), implementation strategy (i.e., switching from circulating medical journal articles to testimonials of respected members of the primary adoption group) or context (i.e., mandating LUS credentialing and use for some clinicians) [28]. Implementation strategies deployed during the 1-year period of data collection as a result of the twice monthly meetings were considered planned adaptations [27–29]. Fidelity consistent adaptations were defined as adaptations that did not increase the likelihood that an inappropriate clinical decision would be made using LUS data based on expert chart review by LUS faculty.

### Data sources

Quantitative data sources for Reach and Adoption included data extracted from the EHR and presented via the RE-AIM dashboard. Fidelity assessment of intervention core components included quality assessment of LUS images using the POCUS imaging archive while image interpretation and clinical decision-making using LUS findings were obtained via expert chart review. Although images are also available for review in the EHR, the imaging archive that contains only hospitalist-performed POCUS exams allowed for easier evaluation of individual hospitalist use. Of note, fidelity assessments (i.e. appraisal of image quality, image interpretation, and clinical decision-making) were performed as part of quality assessment processes required by the hospital as part of routine clinical operations. Data sources for adaptation were extracted from the review of implementation meeting notes.

Qualitative data related to Reach, Adoption, and Implementation were obtained from qualitative hospitalist interviews collected throughout the data collection period as well as meeting notes and implementation team e-mail correspondence documenting the iterative RE-AIM process conducted at the twice monthly project team meetings. Because the PI and implementation team are embedded within their study population as clinical hospitalists, they had frequent conversations with their colleagues regarding hospitalist perceptions of the determinants of LUS. These conversations were shared at twice monthly POCUS meetings and documented in meeting notes. In addition, the research team interviewed 12 hospitalists at different stages of LUS training and adoption representing the full range of use from no experience to regular independent use. The interview guide questions were designed to better understand the determinants of LUS implementation and have been published previously [25]. Emerging themes from these data sources were discussed at the twice monthly project meetings as they became available and influenced implementation strategy selection.

### Data analysis

We report descriptive statistics of the quantitative implementation outcomes of Reach, Adoption, and Implementation and performed a *z*-test when evaluating for differences in subgroups as part of our Reach representativeness assessment. A thematic analysis approach was used to analyze the interview data [30]. Interviews were recorded and professionally transcribed verbatim. Two qualitatively trained members of the research team (AMM and TG) coded qualitative data from the interviews that were specific to COVID. The process began with the team independently reviewing a subset of the transcripts to inductively identify codes and then collaboratively develop a consolidated codebook. After multiple rounds of open coding, the team finalized the codebook, which they applied to the remainder of the transcripts. Transcripts were entered and coded in Dedoose 9.0.17 (SocioCultural Research Consultants LLC, Los Angeles, CA), for data management. The coding process and results of the interview data discussing the general determinants of LUS and not those specific to COVID have been previously described [25].

## Results

### Quantitative data

#### Reach

Over the 1 year of data collection, July 2020 through June 2021, 723 LUS were archived into the EHR, of these 63% (453/723) were used in the clinical care of 298 patients with COVID-19. This was a Reach of 2 % (298/12,567) of the patients with COVID-19 cared for by hospitalists during the 1-year study period. However, there were dynamic changes in the total number of eligible patients and Reach to patients over the study period, with monthly Reach percentages ranging from as low as 0% to as high as 59% (Table 1). With regard to representativeness among eligible patients with a COVID-19 diagnosis, the proportions of patients older than 65 and African-American patients who did not receive LUS compared to those who did were similar. However, a proportionally greater number of Hispanic patients (36% vs 24%; *p*<0.01) and patients who were non-English speaking (27% vs. 14%; *p*<0.01) received LUS than those who did not.

**Table 1:** Study timeline demonstrating dynamic trends in Reach and Adoption

Month/year	Reach (#Patients with COVID who received LUS/total # patients with COVID	Incremental increases in *Credentialing Adoption* (number of new attendings credentialed)	Incremental increases in *Ordering Adoption* (number of new providers ordering)	Implementation strategy deployed or discontinued *(ERIC strategy)*
May 2020	0%(0/255)	0	0	IS 1: Targeted email reminders to COVID faculty. *(Remind clinicians)*
June 2020	1% (1/84)	0	0	
^a^July 2020	28% (35/125)	0	4	IS 2: Mandate procedure service physicians to be credentialed and perform LUS as part of their usual clinical duties (*Mandate change)*
August 2020	59% (37/63)	1 procedure service attending	5	IS 3: Have procedure service attendings supervise and perform image acquisition *(Promote adaptability)*
September 2020	15% (13/89)	0	3	IS 4: Use remote teleguidance software to remotely supervise LUS image acquisition *(Change physical structure and equipment)*
October 2020	2% (4/222)	1 Non-procedure service attending	1	
November 2020	5% (26/547)	0	1	IS 5: Circulate academic papers to address COVID-19-specific barriers (*Distribute educational materials)*
December 2020	1% (10/695)	1 procedure service attending	4	
January 2021	1% (31/2072)	0	2	
February 2021	2% (45/1914)	0	12	IS 6: Intensify IS2 to mandate for procedure service APPs to become credentialed – add accountability metrics and support *(Mandate change)* *IS 1 stopped* *(De-implement)*
March 2021	1% (21/2172)	0	2	
April 2021	2% (35/2295)	1 procedure service attending	2	
May 2021	1% (27/2335)	0	3	IS 7: Billing data accrued demonstrates program is budget neutral and continued funding is approved by clinical leadership *(Access new funding)*
^b^June 2021	1% (32/2147)	0	4	
**Total for the study period**	**2% (298 of 12567)**	**5% (4 of 86)**	50% (43 of 86)	
^c^July 2021	1% (33/2263)	0	0	
^c^August 2021	2% (43/2311)	0	3	
^c^September 2021	3% (70/2348)	0	4	
^c^October 2021	3% (67/2387)	0	8	

^a^Beginning 12-month grant funding period

^b^End 12-month grant funding period

^c^Conducted during the sustainment period

#### Adoption

**Fig. 3:**
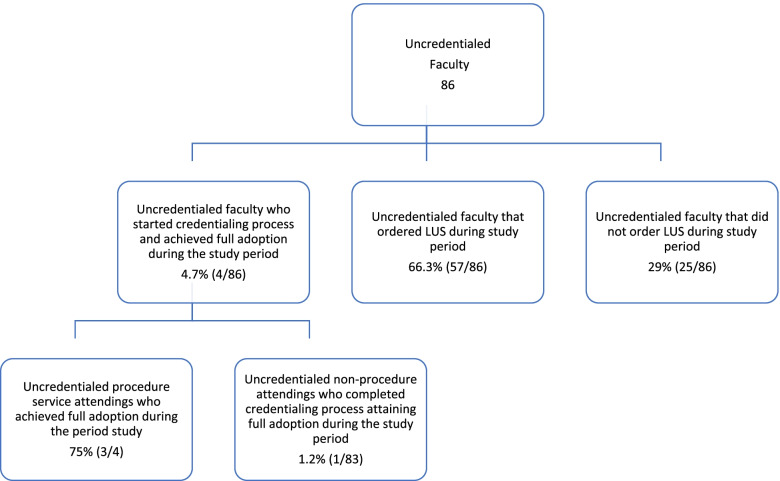
Adoption flowchart

LUSs for patients with COVID were ordered by 43 different faculty members during the 1-year study period but were performed or supervised by only 8 faculty. As discussed below, adoption in this study was complex and consisted of two different measures. As demonstrated in Fig. 1, among the 86 faculty who were not credentialed to perform LUS at the beginning of the study, half of the faculty met the criteria for adoption of ordering LUS (50% adoption rate to *order* LUS), 18 had started their LUS portfolios but only four of the uncredentialed faculty completed the entire training/credentialing process during the study period; all 4 who completed training and obtained credentials performed at least one LUS independently during the study period. This yielded a LUS *credentialing* adoption rate of 1.2% (1 of 83) and 75% (3 of 4) for eligible hospitalists and proceduralists respectively, the latter having a mandate to complete LUS credentialing in contrast to the former that did not have a mandate. We chose to focus on full independent use as our threshold for Adoption as at the beginning of the study there were a very limited number of people who could actually obtain images to fulfill an order, limiting Reach, which is how we arrived at a *credentialing* adoption rate (Fig. 3). Only after introducing an implementation strategy to have proceduralists performing LUS ordered by other hospitalists did we recognize that there was a less complete level of adoption that is ordering a LUS for clinical decision-making. This is how we arrived at our *ordering* adoption rate.

#### Implementation/fidelity

Fidelity was measured via standardized assessment of image quality [21], image interpretation [22], and clinical integration and decision-making via POCUS image archive and chart review by LUS faculty. Ten percent (47 of 453) of randomly selected LUSs obtained in patients with COVID-19 were reviewed for this purpose. Ninety-six percent (45 of 47) of LUS exams reviewed were considered of adequate quality for interpretation. Interpretation and decision-making were determined to be consistent with LUS findings and other clinical data 100% (44 of 44) of the time it was documented in the EMR; however, appropriate documentation was present 91% (44 of 47) of the time.

### Qualitative data from interviews with hospitalists

Between September 30th and December 22nd, 2020, the study team conducted a total of 12 interviews with hospitalists, three of whom had already integrated LUS into their management of patients with and without COVID-19 and nine of whom had not yet adopted LUS. Demographics of interviewees and methods used to understand results of *general barriers* to adoption of LUS captured in these interviews have been described in prior work [25]. The results detailed below arise from the data related to COVID-specific determinants.

Three themes emerged about barriers to adoption of LUS in patients with COVID-19: (1) clinician desire to minimize time in the patient room to decrease risk of contracting COVID-19; (2) the additional time required to obtain LUS due to PPE and additional cleaning of the machines required; and (3) lack of perceived utility and evidence for the use of LUS in the management of COVID-19 patients.

#### Perceived increased risk of infection with the use of LUS in patients with COVID-19

Hospitalists reported a reluctance to spend the additional time necessary to obtain a LUS for fear of increasing the likelihood of contracting COVID-19. Hospitalist A7 said “Many providers want to try to spend as little time in those patient rooms as needed because we're all trying to decrease our own exposure to COVID-19.”

In addition, some clinicians reported not performing LUS because of the worry the ultrasound may act as a fomite. Hospitalist A8 stated: “I feel like there was a decrease in performing ultrasound just because there was so much concern about really anything coming into contact with anyone.”

#### Additional clinician time required to obtain LUS exams in patients with COVID-19

Aside from the perceived increased risk of contracting COVID-19 from spending additional time in the room, hospitalists also reported the additional time spent cleaning the equipment to reduce the risk of transmission of the virus to other patients and staff was a deterrent to obtaining an LUS in COVID-19 patients. Hospitalist A18 said: “The thought of bringing in a probe, the ultrasound machine, and knowing I had to clean it thoroughly before and after every time I used it in a COVID-19 room, it adds up, especially if you’re doing that for multiple patients*.*” Hospitalist 15 said: “For the COVID patients I’ve taken care of, I just imagine that using one ultrasound requires a lot more cleaning.”

#### Lack of evidence and lack of perceived utility

Finally, there were some hospitalists who reported a lack of perceived benefit regarding LUS in the patients with COVID-19. They believed imaging was not necessary to rule out other etiologies because they were expected to decompensate from progression of their COVID-19 pneumonia. Hospitalist A26 was quoted as saying: “We expect patients, especially if they’re in that window, to get worse … I don’t know that we need imaging to confirm that.”

#### Summary of findings

Three key findings emerged from the qualitative and quantitative data collected through the use of Iterative RE-AIM (Fig. 1). The first is that in addition to general barriers to LUS adoption described previously, there are specific COVID-19 barriers, including perceived questionable clinical utility in addition to the extra time required to disinfect the LUS equipment. The second key finding is that many hospitalists were more willing to make a clinical decision without LUS than acquire LUS images themselves, similar to the way traditional imaging studies are performed by Radiology. A third key finding which is suggested by the higher rate of adoption among proceduralist hospitalists relative to non-proceduralist hospitalists is that changing the practice context by mandating credentialing and use among a strategically selected group of hospitalists with limited additional responsibilities *may* have increased the likelihood of full adoption of this hospitalist demographic and facilitated implementation. Details of the qualitative and quantitative data supporting these three key findings as well as the specific implementation strategies deployed in response to them are detailed further in Table 2.

**Table 2:** Key findings and implementation strategies deployed to address them using Iterative RE-AIM

Key findings	Quantitative data	Qualitative data	Implementation strategies deployed that addressed key finding *ERIC* [23] *Strategy type*
There are specific COVID-19 barriers to implementation that impact Reach	Thirty-seven percent of patients who received LUS during the data collection period were patients without COVID-19, despite implementation strategies being focused on use in patients with COVID-19	Perceived increased time in the patient’s room, extra time required to disinfect equipment, and perceived lack of evidence of patient benefit were all unique barriers to LUS use in patients with COVID-19	*Distribute educational materials* - Circulate academic studies demonstrating benefits of use of LUS for patients with COVID-19. *Revise professional roles* - Ensure there is someone who can obtain LUS images for clinicians directly caring for patients to overcome COVID-19-specific barriers of perceived increased risk of infection, transition, and time spent
Clinicians are more willing to order and make clinical decisions using LUS images than acquire images themselves	Forty-three hospitalist faculty ordered LUS for their patients during the data collection period, but only 8 hospitalist individuals performed or supervised the acquisition of these LUS exams	Lack of time to train and perform LUS were important general barriers to full adoption [25]	*Train the trainer* Subgroup of hospitalists (procedure attendings) made responsible for acquiring LUSs for other hospitalists
Changing the practice context by mandating credentialing and use among a strategically selected group may increase the likelihood of adoption and implementation.	Of the 4 faculty who completed training during the study period only 1 was not a procedure attending (75% adoption among eligible procedure service attendings vs. 1.2% adoption among non-procedure service attendings)	Lack of time to train and perform LUS were the important general barriers to full adoption and implementation [25]	*Mandate change* - Require credentialing and use of LUS by a strategically selected subgroup of clinicians (i.e., procedure service faculty)

### Temporal implementation of the Iterative RE-AIM process

The Iterative RE-AIM process revealed the most difficult to address of the implementation team’s prioritized RE-AIM outcomes of Reach, Adoption, and Implementation was low adoption at the staff (hospitalist) level. The low rates of adoption were detected throughout the pilot via twice monthly assessments of RE-AIM outcomes using the RE-AIM dashboard. In total, 6 novel implementation strategies were selected to overcome limited adoption, and these strategies were deployed sequentially over the 1-year grant-funded period of data collection, July 2020 to June 2021. Decisions regarding which implementation strategies should be deployed and discontinued throughout this 12-month period were informed by conversations between the implementation team and hospitalist faculty as well as the qualitative interviews of hospitalists as this information became available. We provide a general description of each strategy in Additional file 1 and compare the timing of each strategy’s deployment with the dynamic changes in Reach and Adoption over time in Table 1.

## Discussion

In this 1-year single-center implementation pilot, we employed a relatively new overarching implementation strategy, Iterative RE-AIM. This led to the selection and deployment of multiple additional strategies during the study’s 12-month implementation phase. This approach was associated with improvements in targeted Reach and Adoption, ultimately resulting in the initial integration of LUS into routine clinical care with high measures of fidelity.

The Iterative RE-AIM process used a RE-AIM dashboard (Fig. 2) that provided nearly real-time audit and feedback [23] to the implementers. While an Iterative RE-AIM process has been used in outpatient environments [17], to our knowledge, this is the first study to use this process in an inpatient environment. As inpatient environments are particularly complex and dynamic contexts [31, 32], the implementation team’s ability to access real-time measures of Reach, Adoption, and Implementation outcomes without delays or ongoing effort was important to the feasibility and speed of this process. Another important potential benefit of the Iterative RE-AIM approach is the opportunity to consider which implementation strategies should be discontinued to redirect implementation resources toward more promising strategies. Other researchers seeking effective strategies to enhance implementation of evidence-based interventions in rapidly changing contexts with finite resources to facilitate implementation may also find this approach promising.

This iterative approach was important in addressing the significant barriers to adoption in this context which included barriers specific to COVID-19 detailed in the results section as well as a lack of time for clinicians to train and perform LUSs described in prior published work [33]. Among the planned adaptation implementation strategies was mandated credentialing and use by a specific subgroup of hospitalists (i.e., procedure service attendings) which led to a new option for hospitalists to order a LUS to be completed on their behalf rather than obtaining the images themselves. The success of this approach in facilitating implementation is demonstrated by the fact that while half of the eligible faculty ordered LUS for their patients only 8 had “fully” adopted (i.e., were credentialed to acquire and interpret LUS independently). This strategy ultimately allowed for a sizable increase in the number of patients who received LUS despite only a small increase in full adoption.

Our convergent mixed methods evaluation revealed three key findings. The first finding was that there were specific COVID-19 barriers to LUS adoption, suggesting each indication for LUS may have unique determinants of adoption for different conditions, contexts, and settings that must be understood if enhanced implementation is desired. The second finding, that hospitalists were more willing to make clinical decisions with LUS performed by others than to acquire LUS images themselves, suggests that the process of adoption may unexpectedly include a phase in which clinicians have “cognitively adopted” LUS into their practice (i.e., learned to interpret LUS images and integrate the imaging findings into their clinical decisions) prior to *or instead of* learning how to acquire images themselves. Given our findings that time scarcity is an important barrier to adoption and implementation, this observation may signal that implementation strategies which reduce the initial investment of time required to learn a new skill by mastering one aspect first should be considered by future implementers. A third key finding suggested by the trend in the higher rate of adoption among proceduralist hospitalists relative to non-proceduralist hospitalists is that changing the practice context by mandating credentialing and use among a strategically selected group of hospitalists *may* have increased the likelihood of full adoption of this hospitalist demographic and greatly facilitated implementation. A possible causal relationship should be explored in future studies.

While these results suggest clinicians felt the potential advantages of LUS did not consistently outweigh barriers to use in the management of all patients with COVID-19, it is important to note that the perceived barriers are likely dynamic. For instance, lack of evidence for support of LUS in COVID-19 could change with time as there have been multiple studies demonstrating utility in COVID-19 published since our pilot ended [34–36]. This may allow a more targeted approach to utilize LUS in the COVID-19 patients who would most benefit. Additionally, many of the interviews occurred prior to the vaccine becoming available and during a time when there were more unknowns about transmission risk, therefore the concern of transmitting COVID-19 may be less pronounced now than when these interviews were conducted. Future studies can explore whether these barriers were found in other hospitals and persist over time.

A novel aspect of this study is describing a planned adaptation *to the context* (e.g., mandate to become credentialed) rather than the intervention or implementation strategy to improve “fit” between the intervention and the context. The conceptualization of adaptation as well as its significance and role in implementation is changing [28, 37]. Only recently has it become accepted as an inevitable and even welcome aspect of the process of implementation. Although there are implementation science frameworks that emphasize environmental changes as a means of facilitating behavior change and adoption [38, 39], to our knowledge, the adaptation literature up to this point has primarily described planned adaptations occurring at the level of the intervention or implementation strategy, not at the level of the context [27, 29, 37]. Given the findings of our pilot and the known impact of policy and mandates on implementation, expanding the standard conceptualization of adaptation as occurring at the level of the context may be useful in enhancing implementation.

Notably, these data do not demonstrate a clear increase in Reach in response to changes in implementation strategies deployed. As this was an uncontrolled pilot study, the ability to definitively determine the factors that contributed to low Reach is limited. However, factors that may have contributed to the limited Reach achieved include our patient eligibility criteria being overly broad, the baseline inexperience of the large majority of the hospitalists with LUS, the lack of continuity in hospitalists caring for patients with COVID, and dynamic contextual factors such as high patient censuses or clinician fatigue over the course of the pandemic making it more difficult for clinicians to adopt new behaviors and skills that require additional time and cognitive resources.

Although Reach was markedly limited throughout the study, we did see an increase in the number of eligible patients who received a LUS from baseline as the number of diagnostic LUS performed went from 1 to 298 over the course of 12 months. This absolute increase in the number of LUSs within a 12-month period suggests employment of the overarching Iterative RE-AIM strategy facilitated implementation. An additional observation arising from these data worth noting is that dynamic changes in Reach reported simply as a percentage may be of limited utility as an iterative measure when implementation is in an early stage and there are large fluctuations in the number of eligible patients. In these cases, transparently reporting the actual number of individuals that received the intervention and the number of those eligible in addition to the percentage, as in Table 1, may be a better way to report progress in implementation.

### Strengths and limitations

This study has several strengths as well as some limitations. In terms of strengths, this study expands the literature on the use of Iterative RE-AIM and using an innovative EHR dashboard application that produced almost real-time data, allowing much more rapid and frequent adaptations. Other strengths include the use of a convergent mixed methods approach to help to understand the barriers to Reach, Adoption and Implementation.

There are also limitations, including that this was a non-randomized, single-center pilot performed in a high-resource academic hospital, thus limiting the generalizability of the results. Additionally, in the chaotic and dynamic context of delivering inpatient care during the COVID-19 pandemic, the implementation team’s process of selecting implementation strategies was not performed according to a formal multi-perspective consensus-building process as recommended in the iterative RE-AIM guidance (www.re-aim.org) [40], and as a result, the implementation strategy selection process may have been biased, favoring the perspective of the PI over that of the other members of the implementation team. In future studies, we plan to use a formal consensus-building process and systematically record the rationale for these decisions using both a checklist and records of project meeting minutes. A third limitation is that this was a pilot study and therefore while we can comment on our perceptions of the feasibility of the Iterative RE-AIM process, it was not compared with another approach in the current study. Future work should compare it to alternative implementation science approaches or quality improvement tools like Plan-Do-Study-Act cycles [41] to see if the perceived advantages of this approach can be demonstrated using rigorous evaluation and objective metrics. Finally, while the representativeness data on Reach suggested some differences in implementation based on ethnicity and non-English speaking status, the implications of these suggested differences are unclear but should be further explored in future studies in other settings with larger sample sizes.

## Conclusions

Adoption of LUS by hospitalists for the management of COVID-19 is limited by the general barrier of clinician time scarcity, in addition to barriers specific to COVID-19 that centered around infection control concerns and perceived utility of LUS use in patients with COVID-19. The Iterative RE-AIM process used in conjunction with a RE-AIM dashboard allowed for the rapid iteration of new implementation strategies in the face of shifting barriers to implementation. Given the dynamic nature of the COVID-19 pandemic, future studies should assess whether these barriers persist over time and are replicated in other hospitals and settings.

## Supplementary Information

**Additional file 1.** Description of Planned Adaptation Implementation Strategies.

## Data Availability

The datasets analyzed during the current study are available from the corresponding author on reasonable request.
