# Supplementary material for: Using Iterative RE-AIM to enhance hospitalist adoption of lung ultrasound in the management of patients with COVID-19: an implementation pilot study
Source: Implement Sci Commun. 2022 Aug 12;3:89. doi: 10.1186/s43058-022-00334-x (PMC9372925; doi:10.1186/s43058-022-00334-x)
Supplement: Supplementary file 1 — Additional file 1. Description of Planned Adaptation Implementation Strategies. [file 43058_2022_334_MOESM1_ESM.docx]

Appendix 1: Description of Planned Adaptation Implementation Strategies

Strategy 1 (IS1 in Table 1) – Remind clinicians/raise awareness: In May 2020, the implementation team targeted clinician awareness of LUS in the management of COVID-19 by emailing hospitalists as they began their COVID-19 service weeks to provide information regarding the study and inviting them to reach out if they desired supervision in performing LUS for their patients with COVID as part of the training and credentialing process. It was quickly realized through twice monthly RE-AIM dashboard inspections and project meetings that adoption remained low and hospitalist responses to these emails were limited, at which point alternative implementation strategies were formulated.  *Target RE-AIM outcome: Adoption*

Strategy 2 (IS2 in Table 1) – Mandate change/new policy: Through discussions with clinical and procedure service leadership a new policy was implemented mandating that hospitalists working on the inpatient procedure service were expected to become credentialed in LUS to improve the supervised scanning opportunities for hospitalist faculty. This new policy change was announced to procedure service attendings via email in July 2020. *Target RE-AIM outcome: Adoption*

Strategy 3 (IS3 in Table 1) – Promote adaptability/alternate form of adoption – ordering LUS: In August 2020, the implementation team created a new opportunity for faculty to adopt by ordering an LUS imaging study to be performed by the procedure service, rather than faculty acquiring the LUS images themselves. Emails were sent out to faculty making them aware that they could now order an LUS for their patients to be performed by the procedure service attendings. This strategy was designed to provide procedure attendings with opportunities to complete their image portfolios (the most time intensive aspect of the credentialing prerequisites), while also increasing Reach despite the small number of clinicians credentialed. *Target RE-AIM outcome: Reach*

Strategy 4 (IS4 in Table 1) – remote teleguidance/supervision of LUS procedure - In September 2020, to improve access to supervised scanning for procedure attendings, LUS supervision via teleguidance software was introduced which enabled remote supervision. This increased the efficiency of implementation efforts while conserving the most limited implementation resource: faculty time to teach LUS. *Target RE-AIM outcomes: Reach and Adoption*

Strategy 5 (IS 5 in Table 1) – Distribute educational materials/influence perceptions of appropriateness - In November of 2020, after hearing from hospitalists through qualitative interviews that an important barrier to adoption of LUS in patients with COVID was a lack of published research demonstrating advantages of LUS specifically in patients with COVID, the implementation team began circulating published literature to address this barrier. *Target RE-AIM outcomes: Reach and Adoption*

Strategy 6 (IS6 in Table 1) – Intensify accountability to the LUS credentialing policy mandate for proceduralists and de-implement strategy 1 - By February of 2021, through frequent inspections of the RE-AIM dashboard and discussions at project meetings, the implementation team reached consensus that focusing efforts on training proceduralists was more likely to result in credentialing than efforts focused on credentialing hospitalists on the COVID services (IS 6,Table 1). Implementation strategies were adjusted to reflect this and implementation resources redistributed from strategy 1 to instead focus more of the implementation team’s most limited resource — faculty time — to training proceduralists, including proceduralist advanced practice providers (APPs) who had recently been added to the procedure service faculty. This messaging included setting clear expectations for completion of credentialing requirements and a timeline for completion which had not been done prior. *Target RE-AIM outcomes: Reach and Adoption*

Strategy 7 (IS7 in Table 1) – Access new funding - In June 2021, at the end of the grant funded project period, the implementation team negotiated with clinical leadership for continued faculty salary support to continue the program, demonstrating via the billing data for LUS acquired over the year-long pilot that this investment would be budget neutral to the DHM. Table 1 demonstrates Reach and Adoption data for the first 4 months of the sustainment period. LUS in the management of COVID was implemented initially in 2020 based on practical concerns regarding the possibility of constrained provider protective equipment resources and potential nosocomial spread of COVID—as these contextual factors were of less concern to hospital leaders later in the pandemic, there was a diminished need to seek further improvements in Reach at the end of the 12-month pilot. Instead, LUS implementation efforts turned to increase the Reach of LUS in the management of heart failure and other disease processes that have even more evidence supporting its use^9^. *Target RE-AIM outcomes: Reach and Adoption*
